# Supplementary material for: Impact of COVID-19 pandemic on acute stroke admissions and case-fatality rate in lower-income and middle-income countries: a protocol for systematic review and meta-analysis
Source: BMJ Open. 2022 Apr 25;12(4):e057893. doi: 10.1136/bmjopen-2021-057893 (PMC9039155; doi:10.1136/bmjopen-2021-057893)
Supplement: Supplementary data [file bmjopen-2021-057893supp001.pdf]

Table 1: Search strings for electronic database

Search #	Searches
1.	Impact OR effect OR influence
2.	"COVID-19"[Mesh] OR "2019-nCoV"[Mesh] OR "severe acute respiratory syndrome coronavirus 2"[Mesh] OR "SARS-CoV-2"[Mesh] OR "Wuhan coronavirus" OR 2019 novel coronavirus* OR ‘’coronavirus disease 2019 virus’’* OR ‘’COVID19 virus’’* OR ‘’2019-nCoV infection’’ OR ‘’novel coronavirus pneumonia’’*
3.	Stroke OR cerebrovascular accident OR CVA OR cerebral infarction OR Ischemic stroke OR Lacuna stroke OR cerebral hemorrhage OR haemorrhagic stroke
4.	Mortality OR death OR case- fatality
5.	Asia OR Africa OR Caribbean OR ‘’Central America’’ OR ‘’South America’’
6.	low- and middle-income countr* OR low and middle income countr* OR low- and middle-income nation*OR low and middle income nation* OR low- and middle-income world OR low and middle income world OR low- and middle-income econom* OR low and middle income econom*OR low income countr* OR middle income countr* OR low-income countr* OR middle-income countr*OR low income nation* OR middle income nation* OR low-income nation* OR middle-income nation* OR low income world OR middle income world OR low-income world OR middle-income world OR low income econom*OR middle income econom* OR low-income econom* OR middle-income econom*
7.	((((((((Afghanistan* OR Benin* OR Burkina Faso* OR Burundi* OR Central African

	Republic* OR Chad* OR Comoros* OR Congo* OR Eritrea* OR Ethiopia* OR Gambia*OR Guinea-Bissau* OR Haiti* OR Korea Republic* OR Liberia* OR Madagascar* OR Malawi* OR Mali* OR Mozambique* OR Nepal* OR Niger* OR Rwanda* OR Sierra Leone* OR Somalia* OR South Sudan* OR Syrian Arab Republic* OR Tajikistan* OR Tanzania* OR Togo* OR Uganda* OR Yemen* OR Zimbabwe* OR Angola* OR Bangladesh* OR Bhutan* OR Bolivia* OR Cabo Verde* OR Cambodia* OR Cameroon*OR Congo* OR Ivory Coast* OR Djibouti* OR Egypt* OR El Salvador* OR Georgia* OR Ghana* OR Honduras* OR India* OR Indonesia* OR Kenya* OR Kiribati* OR Kosovo* OR Kyrgyz Republic* OR Lao PDP* OR Lesotho* OR Mauritania* OR Micronesia* OR Moldova* OR Mongolia* OR Morocco* OR Myanmar* OR Nicaragua* OR Nigeria* OR Pakistan* OR Papua New Guinea* OR Philippines* OR Sao Tome) and Principe*) OR Solomon Islands* OR Sri Lanka* OR Sudan* OR Swaziland* OR Timor-Leste* OR Tunisia* OR Ukraine* OR Uzbekistan* OR Vanuatu* OR Vietnam* OR West Bank) AND Gaza*) OR Zambia* OR Albania* OR Algeria* OR American Samoa* OR Armenia* OR Azerbaijan* OR Belarus* OR Belize* OR Bosnia) AND Herzegovina*) OR Botswana* OR Brazil* OR Bulgaria* OR China* OR Colombia* OR Costa Rica* OR Cuba* OR Dominica* OR Dominican Republic* OR Equatorial Guinea* OR Ecuador* OR Fiji* OR Gabon* OR Grenada* OR Guatemala* OR Guyana* OR Iran* OR Iraq* OR Jamaica* OR Jordan* OR Kazakhstan* OR Lebanon* OR Libya* OR Macedonia* OR Malaysia* OR Maldives* OR Marshall Islands* OR Mauritius* OR Mexico* OR Montenegro* OR Namibia* OR Nauru* OR Paraguay* OR Peru* OR Romania* OR Russian Federation* OR Samoa* OR Serbia* OR South Africa* OR Saint Lucia* OR Saint Vincent) AND the Grenadines*) OR Suriname* OR Thailand* OR Tonga* OR Turkey* OR Turkmenistan* OR Tuvalu* OR Venezuela*).mp.
8.	1 AND 2 AND 3 AND 4 AND 5 AND 7
9.	Limit 6 to humans

Ackah M, *et al. BMJ Open* 2022; 12:e057893. doi: 10.1136/bmjopen-2021-057893
