# Supplementary material for: Impact of COVID-19 pandemic on acute stroke admissions and case-fatality rate in lower-income and middle-income countries: a protocol for systematic review and meta-analysis
Source: BMJ Open. 2022 Apr 25;12(4):e057893. doi: 10.1136/bmjopen-2021-057893 (PMC9039155; doi:10.1136/bmjopen-2021-057893)
Supplement: Author's manuscript [file bmjopen-2021-057893.draft_revisions.pdf]

BMJ Open is committed to open peer review. As part of this commitment we make the peer review history of every article we publish publicly available.

When an article is published we post the peer reviewers' comments and the authors' responses online. We also post the versions of the paper that were used during peer review. These are the versions that the peer review comments apply to.

The versions of the paper that follow are the versions that were submitted during the peer review process. They are not the versions of record or the final published versions. They should not be cited or distributed as the published version of this manuscript.

BMJ Open is an open access journal and the full, final, typeset and author-corrected version of record of the manuscript is available on our site with no access controls, subscription charges or pay-per-view fees (http://bmjopen.bmj.com).

If you have any questions on BMJ Open's open peer review process please email info.bmjopen@bmj.com

BMJ Open

A protocol for systematic review and meta-analysis for impact of COVID-19 pandemic on acute stroke admission and case-fatality in Lower- and Middle-Income Countries.

Journal:	*BMJ Open*
Manuscript ID	bmjopen-2021-057893
Article Type:	Protocol
Date Submitted by the Author:	29-Sep-2021
Complete List of Authors:	Ackah, Martin; Korle Bu Teaching Hospital, Physiotherapy; University of Ghana, School of Public Health, Department of Epidemiology and Disease Control<br>Salifu, Mohammed Gazali; Ministry of Health, Monitoring and Evaluation Directorate; University of Ghana, School of Public Health, Department of Epidemiology and Disease Control<br>Ameyaw, Louise; University of Ghana, School of public Health, Department of Epidemiology and Disease Control<br>Boakye, Hosea; University of Ghana, School of Public Health, Department of Biological, Environmental, and Occupational Health<br>Yeboah, Cynthia Osei; Korle Bu Teaching Hospital, Department of Physiotherapy
Keywords:	COVID-19, NEUROLOGY, Stroke < NEUROLOGY, EPIDEMIOLOGY

SCHOLARONE™
Manuscripts

A protocol for systematic review and meta-analysis for impact of COVID-19 pandemic on acute stroke admission and case-fatality in Lower- and Middle-Income Countries.

Martin Ackah[1, 3], Mohammed Gazali Salifu[2,3], Louise Ameyaw[3], Hosea Boakye[4], Cynthia Osei Yeboah[1]

1. Department of Physiotherapy, Korle Bu Teaching Hospital, Accra, Ghana
2. Monitoring and Evaluation Directorate, Ministry of Health, Accra, Ghana.
3. Department of Epidemiology and Disease Control, School of Public Health, University of Ghana, Accra, Ghana
4. Department of Biological, Environmental, and Occupational Health, School of Public Health, University of Ghana Accra, Ghana

Correspondence to Martin Ackah; martinackah10@gmail.com

For peer review only

For peer review only - http://bmjopen.bmj.com/site/about/guidelines.xhtml

Abstract

Introduction. The current review primarily aims to ascertain the impact of Coronavirus Disease-19 (COVID-19) on stroke admission as well as stroke case-fatality in Low-Middle Income Countries (LMICs).

Method: Four international databases [PubMed/Medline, Google Scholar, African Journals Online (AJOL), Latin American and Caribbean Health Sciences Literature (LILACS)] and one preprint database [medRxiv] will be searched for relevant studies on the impact of COVID-19 pandemic on stroke admissions and/or stroke case fatality in LMICs from December, 2019 to date, without language restriction. Quality assessment will be done by two independent reviewers. The characteristics of the studies will be presented and summarized in a Table. We aim to perform meta-analyses on pooled proportional change in stroke admissions and case-fatality with 95%CI using a random-effects meta-analysis. Publication bias will be assessed using funnel plot and Egger's regression test if ≥10 studies are involved. A sub group analysis will be performed to determine the sources of heterogeneity based on the following, Economic Status (Low-income country and Middle-income country), Continent (Africa, South America, Asia, and Europe] and year of publication. Leave one out sensitivity analysis will be performed to examine the impact of a single study on the overall pool estimate.

Ethics and dissemination: Ethical approval is not required as this is secondary research and will use reported data in scientific literature. A full manuscript will be submitted to a reputable peer-review journal for publication

PROSPERO registration number: CRD42021281580

Strength ad limitation of this study

- This is the first systematic review and meta-analysis on the impact of Covid-19 pandemic on acute stroke admission and case-fatality in LMICs
- A well-validated systematic review and meta-analysis methods that are fully compliant with established international norms and guidelines would be followed.
- To examine the robustness of the derived estimates, a sensitivity and sub-group analysis would be done.
- There would be no language restriction
- As a result of the regional and cultural variances, there would be significant heterogeneity across the studies.

Keywords: Impact; Stroke admission; Case fatality; COVID-19; LMICs

For peer review only

Introduction

The World Health Organization (WHO) announced a Severe Acute Respiratory Syndrome Coronavirus-2 (SARS Cov-2) pandemic in March 2020, posing a threat to healthcare systems and cultures around the world [1]. Since the first recorded case in December of 2019, more than 240 million Coronavirus Disease-19 cases and 4.7 million deaths have been confirmed worldwide as of September 25, 2021, with Africa accounting for approximately 3% of global incidence[2]

COVID-19 is largely a respiratory infection, but research in patients with severe infections has showed that it is a multi-system inflammatory condition with neurologic effects such as stroke [3]. For example, stroke rates among confirmed and hospitalized COVID-19 patients have been found to range from 1.4% to 1.74% according to recent global systematic review and meta-analyses [4,5].

There have been conflicting accounts on the impact of stroke admission appearing in emergency rooms around the High-Income Countries (HIC) and Lower-and Middle-Income countries (LMICs) during the pandemic. For instance, available observational data showed that stroke admission declined by 12% in United Kingdom [6], 23% in Spain [7], 30% in France [8] , 36% in Brazil[9], 40% in China [1], and whooping 50% reduction in Bangladesh[10]. However, stroke admission during the pandemic increased by 42% in Abu Dhabi[11], 4% and 21% in Italy[12,13]. Furthermore, a recent observational study in Sub-Sahara Africa (SSA) found that the number of stroke admissions increased by approximately 8% in the first half of 2020 compared to the same time in 2019, [14].

Despite documented high-quality services, the pandemic has had a negative impact on stroke case fatality in both HIC and LMICs based on observational studies; stroke case fatality increased by 2.4 percentage points in the United Kingdom [6], and by a significant 60 percent in France [8]. A cross

For peer review only - http://bmjopen.bmj.com/site/about/guidelines.xhtml

sectional study in SSA found an estimated peri-pandemic high stroke case fatality rates of 29.3% [14].

A global systematic review and meta-analysis on the impact of covid-19 pandemic on stroke admission found a significant reduction of 29% compared to pre-pandemic period[15]. The authors did not conduct a sub-group analysis to determine the influence of the pandemic on stroke admission and case fatality in LMICs. Furthermore, just a few papers from LMICs were included in the prior review. Obviously, this cannot be generalized to represent covid-19 impact of stroke admissions and its case fatality in LMICs'

Though observational studies on covid-19 impact on stroke admission and case-fatality have been conducted, no prior systematic review and meta-analysis has been undertaken in LMICs. With this in mind, the authors sort to conduct a systematic review and meta-analysis on the impact of covid-19 pandemic on acute stroke hospitalization and death in limited-resource countries in the world.

Research questions

1. What is the impact of COVID-19 pandemic on stroke admission in LMICs?
2. What is the impact of the COVID-19 pandemic on stroke case fatality in LMICs?

For peer review only

For peer review only - http://bmjopen.bmj.com/site/about/guidelines.xhtml

Method

Patients and public involvement

Patient or the public were not involved in the design, or conduct, or reporting, or dissemination plans of our research

Ethics and dissemination

Ethical approval is not required as this is secondary research and will use reported data in scientific literature. A full manuscript will be submitted to a reputable peer-review journal for publication.

Best practices and registration

The current protocol was registered in the PROSPERO database under the identifier CRD42021281580, and it was reported in compliance with Preferred Reporting Items for Systematic Review and Meta-analyses Protocol (PRISMA-P)[16] [checklist file].

Eligibility criteria

Studies

Observational studies such as longitudinal, cohort, case control and cross-sectional studies reporting the impact of COVID-19 pandemic on stroke admission and case fatality in LMICs will be considered in the current review. Case series, case reports, narrative and systematic reviews, editorials, comments, letters, opinion pieces, abstracts, conference proceedings, and animal studies will all be excluded.

Participants

We aim to include Impact of COVID-19 pandemic on stroke admission and case fatality studies involving all age group from LMICs. General medical admission and studies from HIC will be excluded. LMICs are countries defined by World Bank Group [17]

For peer review only - http://bmjopen.bmj.com/site/about/guidelines.xhtml

Intervention/exposure

Exposure is the COVID-19 pandemic

Comparators

The study will compare rates of stroke admissions and case fatality between pre-COVID-19 pandemic era and peri/post-COVD-19 pandemic era

Outcome of interest

The outcome of interest will be; (1) the impact of COVID-19 pandemic on stroke admission in LMICs. (2) the impact of COVID-19 pandemic on stroke case fatality in LMICs

Data source and search strategies

Four international databases [i.e., PubMed/Medline, Google Scholar, African Journals Online (AJOL), Latin American and Caribbean Health Sciences Literature (LILACS)] and one preprint database [medRxiv] will be searched for relevant studies on the impact of COVID-19 pandemic on stroke admissions and/or stroke case fatality in LMICs from December, 2019 to date, without language restriction.

Medical Sub-Heading (MeSH) terms and free text will be used in the search approach. These terms will be combined with the Boolean operators ‘OR' and ‘AND'. The key term includes; Covid-19, Stroke, cerebrovascular accident, admissions, case fatality, Low- and middle-income countries, Africa, Sub-Saharan Africa, Asia, Latin America, Caribbeans. The full search string is shown in **supplementary file 1**

For peer review only

For peer review only - http://bmjopen.bmj.com/site/about/guidelines.xhtml

Other sources

The authors will evaluate the reference lists of papers that meet the inclusion and exclusion criteria to see whether there will be any additional studies that are luckily to be missed by our electronic search.

Data screening and selection

MA and LA will independently review the titles and abstracts against the pre-specified eligibility criteria for inclusion in the review, and then repeat the process after retrieving the full-text. A disagreement will be resolved by discussion or reference to a third reviewer

Data extraction and management

The data extraction will be done by two researchers independently using a pretested and standardized excel spreadsheet[18]. Discrepancies arising during abstract and full text review will be discussed and agreement reached by adjudication of a third author. Data such as the author's name, year of publication, country, study design, patients' characteristics {i.e., sex, age], pre-pandemic stroke admission and case fatality, peri-pandemic stroke admission and case fatality, total sample size will be extracted. Mendeley will be used to managed and remove duplicated articles.

Addressing missing data

If any of the selected articles contain inadequate information, we will contact the appropriate author through email to request missing data. If that is not possible, the data will be deleted.

For peer review only - http://bmjopen.bmj.com/site/about/guidelines.xhtml

Furthermore, the authors will obtain raw data from the primary study authors wherever possible so that we can extract the missing data

Quality assessment and risk of bias

The Critical Appraisal Checklist for Analytical Cross-Sectional Studies from the Joanna Briggs Institute will be used for the quality assessment and risk of bias[19]. The quality assessments and bias risk assessments will be carried out by two independent peer reviewers. Discrepancies discovered during the abstract and full text reviews will be explored and resolved through discussion. To reduce selection bias, reviewers will be blinded to the article's author, journal, and year of publication during the title and abstract review.

Data synthesis

Extracted data will be exported into Stata (version 16; Stata Cooperation, TX, USA) from Microsoft excel 2013 for all analyses. The study selection procedure will be summarized using the 2020 PRISMA flowchart[20] (Figure 1) The characteristics of the studies will be presented and summarized in a Table. We aim to perform meta-analyses on pooled proportional change in stroke admissions and case-fatality with 95%CI using a random-effects model. The heterogeneity of effect size estimates across these studies will be quantified using the I^2 statistic and Q statistic's P-value[21]. Publication bias will be assessed using funnel plot and Egger's regression test if ≥10 studies are involved.

Where significant heterogeneity exists in the included studies, a sub group analysis will be performed to determine the sources of heterogeneity based on the following, Economic Status (Low-income country and Middle-income country), Continent (Africa, South America, Asia] and

For peer review only - http://bmjopen.bmj.com/site/about/guidelines.xhtml

year of publication. Leave one out sensitivity analysis will be performed to examine the impact of a single study on the overall pool estimate.

For peer review only

For peer review only - http://bmjopen.bmj.com/site/about/guidelines.xhtml

Expected key results and discussion

The impact of covid-19 on stroke admission and mortality in some LMICs have been examined through observational studies with conflicting results[10,14]. Furthermore, no prior systematic review and meta-analysis on this critical topic has been conducted in LMICs. As a result, the current review primarily aims to ascertain the impact of covid-19 on stroke admission as well as stroke case-fatality in LMICs.

For peer review only

For peer review only - http://bmjopen.bmj.com/site/about/guidelines.xhtml

Acknowledgement

None

Contributors

MA conceived the study, drafted the manuscript, critically revised the manuscript for methodological, and intellectual content. MGS drafted the manuscript, critically revised the manuscript for methodological and intellectual content. LA critically revised the manuscript for methodological and intellectual content. HB drafted the manuscript, critically revised the manuscript for methodological and intellectual content. COY drafted the manuscript, critically revised the manuscript for methodological and intellectual content. All authors approved the final manuscript. MA is the guarantor of the review

Funding

The authors have not declared a specific grant for this research from any funding agency in the public, commercial or not-for-profit sectors

Competing interest

The authors declare that there are no conflicts of interest

Patient consent for publication: Not required

Provenance and peer review

Not commissioned; externally peer-reviewed.

Data availability statement

All relevant data are within the manuscript

For peer review only - http://bmjopen.bmj.com/site/about/guidelines.xhtml

Reference

1. Zhao J, Li H, Kung D, Fisher M, Shen Y, Liu R. Impact of the COVID-19 Epidemic on Stroke Care and Potential Solutions. *Stroke*. 2020;(July):1996-2001. doi:10.1161/STROKEAHA.120.030225

2. WHO. *WHO. WHO African Region Numbers at a Glance. 2020; Available from: Https://Www.Afro.Who.Int/Health-Topics/Coronavirus-Covid-19.*; 2021.

3. Markus HS, Brainin M. COVID-19 and stroke—A global World Stroke Organization perspective. *Int J Stroke*. 2020;15(4):361-364. doi:10.1177/1747493020923472

4. Nannoni S, de Groot R, Bell S, Markus HS. Stroke in COVID-19: A systematic review and meta-analysis. *Int J Stroke*. 2021;16(2):137-149. doi:10.1177/1747493020972922

5. Siow I, Lee KS, Zhang JJY, Saffari SE, Ng A, Young B. Stroke as a Neurological Complication of COVID-19: A Systematic Review and Meta-Analysis of Incidence, Outcomes and Predictors: Stroke and COVID-19. *J Stroke Cerebrovasc Dis*. 2021;30(3):105549. doi:10.1016/j.jstrokecerebrovasdis.2020.105549

6. Douiri A, Muruet W, Bhalla A, et al. Stroke Care in the United Kingdom during the COVID-19 Pandemic. *Stroke*. 2021;(June):2125-2133. doi:10.1161/STROKEAHA.120.032253

7. Rudilosso S, Laredo C, Vera V, et al. Acute Stroke Care Is at Risk in the Era of COVID-19: Experience at a Comprehensive Stroke Center in Barcelona. *Stroke*. 2020;(July):1991-1995. doi:10.1161/STROKEAHA.120.030329

For peer review only

For peer review only - http://bmjopen.bmj.com/site/about/guidelines.xhtml

8. Gabet A, Grave C, Tuppin P, Chatignoux E, Béjot Y, Olié V. Impact of the COVID-19 pandemic and a national lockdown on hospitalizations for stroke and related 30-day mortality in France: A nationwide observational study. *Eur J Neurol*. 2021;28(10):3279-3288. doi:10.1111/ene.14831

9. Diegoli H, Magalhães PSC, Martins SCO, et al. Decrease in Hospital Admissions for Transient Ischemic Attack, Mild, and Moderate Stroke during the COVID-19 Era. *Stroke*. 2020;51(8):2315-2321. doi:10.1161/STROKEAHA.120.030481

10. Hasibul Hasan ATM, Das SC, Islam MS, et al. Impact of COVID-19 on hospital admission of acute stroke patients in Bangladesh. *PLoS One*. 2021;16(1 January):1-9. doi:10.1371/journal.pone.0240385

11. John S, Irteza S, Piechowski-jozwiak B, et al. Clinical characteristics and admission patterns of stroke patients during the COVID 19 pandemic : A single center retrospective , observational study from the Abu Dhabi , United Arab Emirates. *Clin Neurol Neurosurg*. 2020;199(106227).

12. Zini A, Romoli M, Gentile M, et al. The stroke mothership model survived during COVID-19 era : an observational single-center study in Emilia-Romagna , Italy. *Neurol Sci*. 2020;41:3395-3399.

13. Frisullo G, Brunetti V, Iorio R Di, et al. Effect of lockdown on the management of ischemic stroke : an Italian experience from a COVID hospital. *Neurol Sci*. 2020.

14. Sarfo FS, Mensah NO, Opoku FA, Adusei-mensah N, Ampofo M, Ovbiagele B. COVID-19 and stroke: Experience in a Ghanaian healthcare system. *J Neurol Sci*. 2020;(January).

For peer review only

For peer review only - http://bmjopen.bmj.com/site/about/guidelines.xhtml

15. Reddy ST, Satani N, Beauchamp JES, et al. A meta-analysis of the global impact of the COVID-19 pandemic on stroke care & the Houston Experience. *Ann Clin Transl Neurol*. 2021;8(4):929–937. doi:10.1002/acn3.51322

16. Moher D, Shamseer L, Clarke M, et al. Preferred reporting items for systematic review and meta-analysis protocols (PRISMA-P) 2015 statement. *Syst Rev*. 2015;(January):1-9.

17. Group WB. Data World Bank Country and Lending Groups. https://datahelpdesk.worldbank.org/knowledgebase/articles/906519-world-bank-country-and-lending-groups. Published 2019. Accessed September 29, 2021.

18. Ackah M, Yeboah CO, Ameyaw L. Risk factors for 30-day in-hospital mortality for in-patient with stroke in sub-Saharan Africa: Protocol for a systematic review and meta-analysis. *BMJ Open*. 2021;11(7):8-12. doi:10.1136/bmjopen-2021-049927

19. JBI. *Checklist for Analytical Cross Sectional Studies*.; 2017.

20. Page MJ, Mckenzie JE, Bossuyt PM, et al. The PRISMA 2020 statement : an updated guideline for reporting systematic reviews Systematic reviews and Meta-Analyses. *BMJ*. 2021. doi:10.1136/bmj.n71

21. Higgins JPT, Thompson SG. Quantifying heterogeneity in a meta-analysis. *Stat Med*. 2002;21(11):1539-1558. doi:10.1002/sim.1186

For peer review only

Figure legend

Figure 1: PRISMA 2020 flow diagram for new systematic reviews which included searches of databases, registers and other sources

For peer review only

For peer review only - http://bmjopen.bmj.com/site/about/guidelines.xhtml

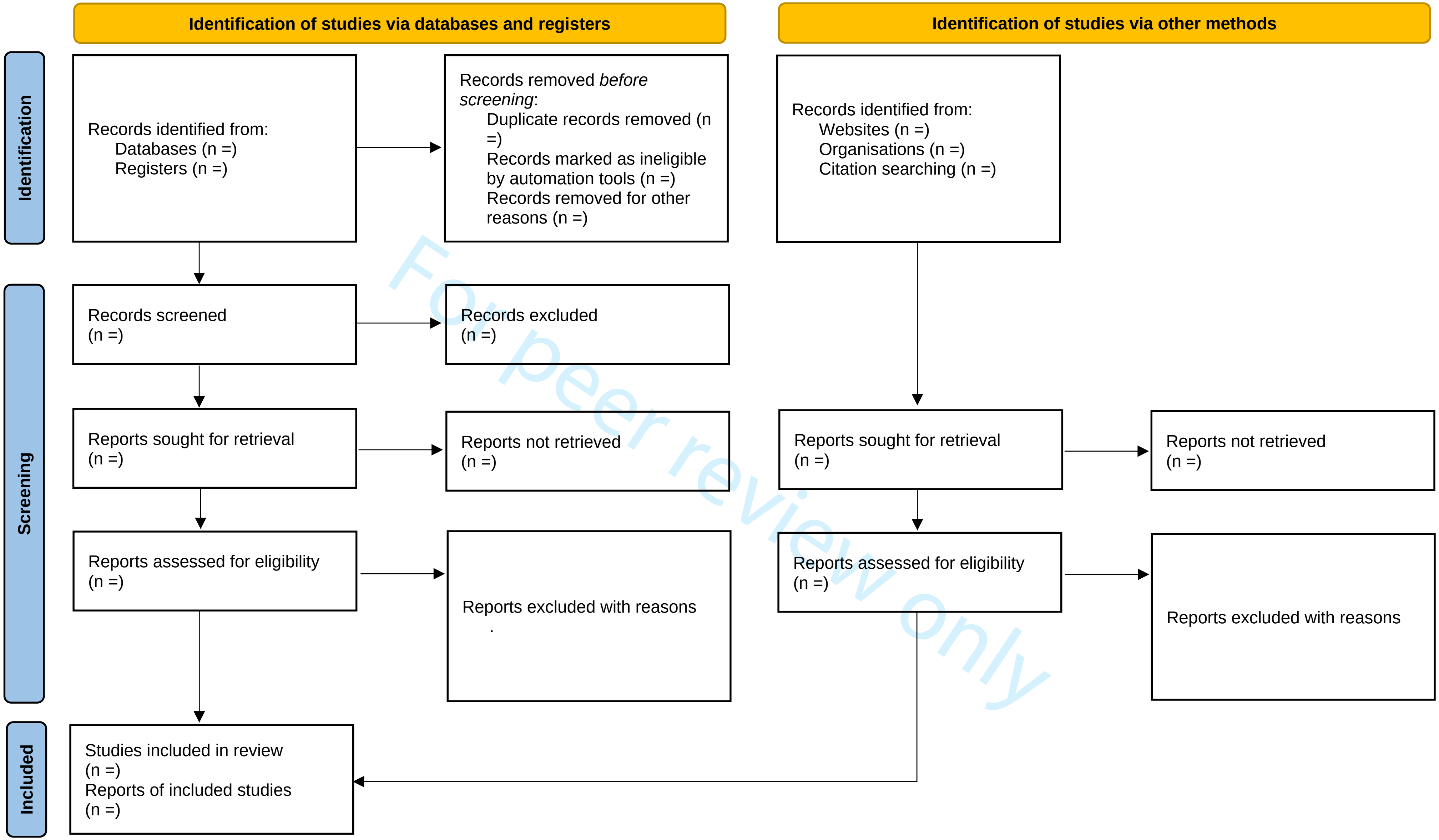

Figure 1: PRISMA 2020 flow diagram for new systematic reviews which included searches of databases, registers and other sources

For peer review only

Table 1: Search strings for electronic database

Search #	Searches
1.	Impact OR effect OR influence
2.	"COVID-19"[Mesh] OR "2019-nCoV"[Mesh] OR "severe acute respiratory syndrome coronavirus 2"[Mesh] OR "SARS-CoV-2"[Mesh] OR "Wuhan coronavirus" OR 2019 novel coronavirus* OR ‘’coronavirus disease 2019 virus’’* OR ‘’COVID19 virus’’* OR ‘’2019-nCoV infection’’ OR ‘’novel coronavirus pneumonia’’*
3.	Stroke OR cerebrovascular accident OR CVA OR cerebral infarction OR Ischemic stroke OR Lacuna stroke OR cerebral hemorrhage OR haemorrhagic stroke
4.	Mortality OR death OR case- fatality
5.	Asia OR Africa OR Caribbean OR ‘’Central America’’ OR ‘’South America’’
6.	low- and middle-income countr* OR low and middle income countr* OR low- and middle-income nation*OR low and middle income nation* OR low- and middle-income world OR low and middle income world OR low- and middle-income econom* OR low and middle income econom*OR low income countr* OR middle income countr* OR low-income countr* OR middle-income countr*OR low income nation* OR middle income nation* OR low-income nation* OR middle-income nation* OR low income world OR middle income world OR low-income world OR middle-income world OR low income econom*OR middle income econom* OR low-income econom* OR middle-income econom*
7.	((((((((Afghanistan* OR Benin* OR Burkina Faso* OR Burundi* OR Central African

For peer review only - http://bmjopen.bmj.com/site/about/guidelines.xhtml

	Republic* OR Chad* OR Comoros* OR Congo* OR Eritrea* OR Ethiopia* OR Gambia*OR Guinea-Bissau* OR Haiti* OR Korea Republic* OR Liberia* OR Madagascar* OR Malawi* OR Mali* OR Mozambique* OR Nepal* OR Niger* OR Rwanda* OR Sierra Leone* OR Somalia* OR South Sudan* OR Syrian Arab Republic* OR Tajikistan* OR Tanzania* OR Togo* OR Uganda* OR Yemen* OR Zimbabwe* OR Angola* OR Bangladesh* OR Bhutan* OR Bolivia* OR Cabo Verde* OR Cambodia* OR Cameroon*OR Congo* OR Ivory Coast* OR Djibouti* OR Egypt* OR El Salvador* OR Georgia* OR Ghana* OR Honduras* OR India* OR Indonesia* OR Kenya* OR Kiribati* OR Kosovo* OR Kyrgyz Republic* OR Lao PDP* OR Lesotho* OR Mauritania* OR Micronesia* OR Moldova* OR Mongolia* OR Morocco* OR Myanmar* OR Nicaragua* OR Nigeria* OR Pakistan* OR Papua New Guinea* OR Philippines* OR Sao Tome) and Principe*) OR Solomon Islands* OR Sri Lanka* OR Sudan* OR Swaziland* OR Timor-Leste* OR Tunisia* OR Ukraine* OR Uzbekistan* OR Vanuatu* OR Vietnam* OR West Bank) AND Gaza*) OR Zambia* OR Albania* OR Algeria* OR American Samoa* OR Armenia* OR Azerbaijan* OR Belarus* OR Belize* OR Bosnia) AND Herzegovina*) OR Botswana* OR Brazil* OR Bulgaria* OR China* OR Colombia* OR Costa Rica* OR Cuba* OR Dominica* OR Dominican Republic* OR Equatorial Guinea* OR Ecuador* OR Fiji* OR Gabon* OR Grenada* OR Guatemala* OR Guyana* OR Iran* OR Iraq* OR Jamaica* OR Jordan* OR Kazakhstan* OR Lebanon* OR Libya* OR Macedonia* OR Malaysia* OR Maldives* OR Marshall Islands* OR Mauritius* OR Mexico* OR Montenegro* OR Namibia* OR Nauru* OR Paraguay* OR Peru* OR Romania* OR Russian Federation* OR Samoa* OR Serbia* OR South Africa* OR Saint Lucia* OR Saint Vincent) AND the Grenadines*) OR Suriname* OR Thailand* OR Tonga* OR Turkey* OR Turkmenistan* OR Tuvalu* OR Venezuela*).mp.
8.	1 AND 2 AND 3 AND 4 AND 5 AND 7
9.	Limit 6 to humans

For peer review only - http://bmjopen.bmj.com/site/about/guidelines.xhtml

For peer review only

For peer review only - http://bmjopen.bmj.com/site/about/guidelines.xhtml

Checklist file 1: PRISMA-P (Preferred Reporting Items for Systematic review and Meta-Analysis Protocols) 2015 checklist: recommended items to address in a systematic review protocol

Section and topic	Item No	Checklist item	Page
**ADMINISTRATIVE INFORMATION**			
Title			
Identification	1a	Identify the report as a protocol of a systematic review	1
Update	1b	If the protocol is for an update of a previous systematic review, identify as such	NA
Registration	2	If registered, provide the name of the registry (such as PROSPERO) and registration number	2 & 6
Authors: contact	3a	Provide name, institutional affiliation, e-mail address of all protocol authors; provide physical mailing address of corresponding author	1
Authors' contribution	3b	Describe contributions of protocol authors and identify the guarantor of the review	11
Amendments	4	If the protocol represents an amendment of a previously completed or published protocol, identify as such and list changes; otherwise, state plan for documenting important protocol amendments	NA
Support			
Sources	5a	Indicate sources of financial or other support for the review	11
Sponsor	5b	Provide name for the review funder and/or sponsor	NA
Role of funder	5c	Describe roles of funder(s), sponsor(s), and/or institution(s), if any, in developing the protocol	NA
**INTRODUCTION**			
Rationale	6	Describe the rationale for the review in the context of what is already known	4-5
Objectives	7	Provide an explicit statement of the question(s) the review will address with reference to participants, interventions, comparators, and outcomes (PICO)	5
**METHODS**			
Eligibility criteria	8	Specify the study characteristics (such as PICO, study design, setting, time frame) and report characteristics (such as years considered, language, publication status) to be used as criteria for eligibility for the review	6-7
Information sources	9	Describe all intended information sources (such as electronic databases, contact with study authors, trial registers or other grey literature sources with planned dates of coverage	7

For peer review only

Search strategy	10	Present draft of search strategy to be used for at least one electronic database, including planned limits, such that it could be repeated	7
Study record			
Data management	11a	Describe the mechanism(s) that will be used to manage records and data throughout the review	8
Selection process	11b	State the process that will be used for selecting studies (such as two independent reviewers) through each phase of the review (that is, screening, eligibility and inclusion in meta-analysis)	8
Data collection process	11c	Describe planned method of extracting data from reports (such as piloting forms, done independently, in duplicate), any processes for obtaining and confirming data from investigators	9
Data items	12	List and define all variables for which data will be sought (such as PICO items, funding sources), any preplanned data assumptions and simplifications	-
Outcome and prioritization	13	List and define all outcomes for which data will be sought, including prioritization of main and additional outcomes, with rationale	7
Risk of bias in the individual studies	14	Describe anticipated methods for assessing risk of bias of individual studies, including whether this will be done at the outcome or study level, or both; state how this information will be used in data synthesis	9
Data synthesis	15a	Describe criteria under which study data will be quantitatively synthesized	9
	15b	If data are appropriate for quantitative synthesis, describe planned summary measures, methods of handling data and methods of combining data from studies, including any planned exploration of consistency (such as I^2, Kendall's τ)	
	15c	Describe any proposed additional analyses (such as sensitivity or subgroup analyses, meta-regression)	9
	15d	If quantitative synthesis is not appropriate, describe the type of summary planned	9
Meta biases	16	Specify any planned assessment of meta-bias(es) (such as publication bias across studies, selective reporting within studies)	9
Confidence in cumulative evidence	17	Describe how the strength of the body of evidence will be assessed (such as GRADE)	-

From: Shamseer L, Moher D, Clarke M, Ghersi D, Liberati A, Petticrew M, et al. Preferred reporting items for systematic review and meta-analysis protocols (prisma-p) 2015: Elaboration and explanation. BMJ [Internet]. 2015;349(January):1–25. Available from: http://dx.doi.org/doi:10.1136/bmj.g7647

For peer review only

For peer review only

BMJ Open

Impact of COVID-19 pandemic on acute stroke admissions and case-fatality rate in Lower- and Middle-Income Countries: a protocol for systematic review and meta-analysis.

Journal:	*BMJ Open*
Manuscript ID	bmjopen-2021-057893.R1
Article Type:	Protocol
Date Submitted by the Author:	28-Feb-2022

Complete List of Authors:	Ackah, Martin; Korle Bu Teaching Hospital, Physiotherapy; University of Ghana, School of Public Health, Department of Epidemiology and Disease Control<br>Salifu, Mohammed Gazali; Ministry of Health, Monitoring and Evaluation Directorate; University of Ghana, School of Public Health, Department of Epidemiology and Disease Control<br>Ameyaw, Louise; University of Ghana, School of public Health, Department of Epidemiology and Disease Control<br>Boakye, Hosea; University of Ghana, School of Public Health, Department of Biological, Environmental, and Occupational Health<br>Yeboah, Cynthia Osei; Korle Bu Teaching Hospital, Department of Physiotherapy

**Primary Subject Heading**:	Global health
Secondary Subject Heading:	Cardiovascular medicine, Epidemiology, Infectious diseases, Neurology, Public health
Keywords:	COVID-19, NEUROLOGY, Stroke < NEUROLOGY, EPIDEMIOLOGY

SCHOLARONE™
Manuscripts

Impact of COVID-19 pandemic on acute stroke admissions and case-fatality rate in Lower- and Middle-Income Countries: a protocol for systematic review and meta-analysis.

Martin Ackah[1, 3], Mohammed Gazali Salifu[2,3], Louise Ameyaw[3], Hosea Boakye[4], Cynthia Osei Yeboah[1]

1. Department of Physiotherapy, Korle Bu Teaching Hospital, Accra, Ghana
2. Monitoring and Evaluation Directorate, Ministry of Health, Accra, Ghana.
3. Department of Epidemiology and Disease Control, School of Public Health, University of Ghana, Accra, Ghana
4. Department of Biological, Environmental, and Occupational Health, School of Public Health, University of Ghana Accra, Ghana

Correspondence to Martin Ackah; martinackah10@gmail.com

For peer review only

Abstract

Introduction. The current review primarily aims to ascertain the impact of Coronavirus Disease-19 (COVID-19) on stroke admission as well as stroke case-fatality in Low-Middle Income Countries (LMICs).

Method: Four international databases [PubMed/Medline, Google Scholar, African Journals Online (AJOL), Latin American and Caribbean Health Sciences Literature (LILACS)] and one preprint database [medRxiv]. Studies will be included if they are conducted in LMICs, all stroke types without age and language restriction, from December, 2019 to 31 December,2021. Two authors will screen the titles and abstracts against the pre-specified eligibility criteria for inclusion in the review, and then repeat the process after retrieving the full-text. Joanna Briggs critical appraisal checklist for analytical cross-sectional studies will be used for the quality assessment and risk of bias by two co-authors. The characteristics of the studies will be presented and summarized in a Table. We aim to perform meta-analyses on pooled proportional change in stroke admissions and case-fatality with 95%CI using a random-effects meta-analysis. Publication bias will be assessed using funnel plot and Egger's regression test if ≥10 studies are involved. A sub group analysis will be performed to determine the sources of heterogeneity. Leave one out sensitivity analysis will be performed to examine the impact of a single study on the overall pool estimate.

Ethics and dissemination: Ethical approval is not required as this is secondary research and will use reported data in scientific literature. A full manuscript will be submitted to a reputable peer-review journal for publication

PROSPERO registration number: CRD42021281580

For peer review only - http://bmjopen.bmj.com/site/about/guidelines.xhtml

Strength ad limitation of this study

- This is the first systematic review and meta-analysis on the impact of Covid-19 pandemic on Acute Stroke Admission (ASA) and Case-fatality Rate (CFR) in LMICs
- A well-validated systematic review and meta-analysis methods that are fully compliant with established international norms and guidelines would be followed.
- To examine the robustness of the derived estimates, a sensitivity and sub-group analysis would be done.
- There would be no language restriction
- As a result of the regional and cultural variances, there would be significant heterogeneity across the studies.

Keywords: Impact; Stroke admission; Case fatality; COVID-19; LMICs

For peer review only - http://bmjopen.bmj.com/site/about/guidelines.xhtml

Introduction

The World Health Organization (WHO) announced a Severe Acute Respiratory Syndrome Coronavirus-2 (SARS Cov-2) pandemic in March 2020, posing a threat to healthcare systems and cultures around the world [1]. Since the first recorded case in December of 2019, more than 240 million Coronavirus Disease-19 cases and 4.7 million deaths have been confirmed worldwide as of September 25, 2021, with Africa accounting for approximately 3% of global incidence[2]

COVID-19 is largely a respiratory infection, but research in patients with severe infections has showed that it is a multi-system inflammatory condition with neurologic effects such as stroke [3]. For example, stroke rates among confirmed and hospitalized COVID-19 patients have been found to range from 1.4% to 1.74% according to recent global systematic review and meta-analyses [4,5].In 2021, the global predicted Case Fatality Rate (CFR) of COVID-19 ranged from 10%[6] to nearly 18%[7].

There have been conflicting accounts on the impact of Acute Stroke Admission (ASA) appearing in emergency rooms. For example, a systematic review and meta-analysis showed that the proportion of haemorrhagic stroke admissions increased in the pandemic period compared to the proportion of haemorrhagic stroke admission in the pre-pandemic era by 10%[8]. The study was limited by several factors; first, the total number of admissions during the pandemic was not reported, rendering the incidence of stroke inconclusive; second, the lack of data about stroke severity, ischemic stroke sub types, and prognoses, might have underestimated/overestimated the pandemic's impact on stroke to some extent[8]. Conversely, a global systematic review and meta-analysis on the impact of COVID-19 pandemic on ASA found a significant reduction of 29% compared to pre-pandemic period[9]. The authors did not conduct a sub-group analysis to determine the influence of the pandemic on ASA in LMICs. Furthermore, just a few papers from LMICs

For peer review only - http://bmjopen.bmj.com/site/about/guidelines.xhtml

were included in the prior review. Obviously, this cannot be generalized to represent the panoramic COVID-19 impact of acute ASA in LMICs.

Despite documented high-quality services with respect to workflow metrics, angiographic results, complications, outcomes at discharge, maintenance of reperfusion therapies as well as infection control measures[10,11], the pandemic has had a negative impact on stroke CFR in both high income countries and LMICs based on observational studies[12–14]. For instance, stroke CFR increased by 2.4 percentage points in the United Kingdom [12], and by a significant 60 percent in France [13]. A cross sectional study in SSA found an estimated peri-pandemic high stroke case fatality rates of 29.3% [14].

Several primary investigations have been carried out to determine the peri-pandemic stroke CFR in LMICs with widely varying reported magnitudes. The authors are unaware of any previous systematic reviews and/or meta-analyses on the subject in LMICs. With this in mind, the authors sort to conduct a systematic review and meta-analysis on the impact of COVID-19 pandemic on acute stroke hospitalization and death in limited-resource countries in the world.

Research questions

1. What is the impact of COVID-19 pandemic on ASA in LMICs?
2. What is the impact of the COVID-19 pandemic on post-stroke CFR in LMICs?

Method and analysis

Ethics and dissemination

Ethical approval is not required as this is secondary research and will use reported data in scientific literature. A full manuscript will be submitted to a reputable peer-review journal for publication.

For peer review only - http://bmjopen.bmj.com/site/about/guidelines.xhtml

Patients and public involvement

Patient or the public were not involved in the design, or conduct, or reporting, or dissemination plans of our research

Best practices and registration

The current protocol was registered in the PROSPERO database under the identifier CRD42021281580, and it was reported in compliance with Preferred Reporting Items for Systematic Review and Meta-analyses Protocol (PRISMA-P)[15] [checklist file].

Eligibility criteria

Studies

Observational studies such as longitudinal, cohort, case control and cross-sectional studies reporting the impact of COVID-19 pandemic on stroke admission and case fatality in LMICs will be considered in the current review. Case series, case reports, narrative and systematic reviews, editorials, comments, letters, opinion pieces, abstracts, conference proceedings, and animal studies will all be excluded.

Participants

We aim to include impact of COVID-19 pandemic on ASA and CFR studies involving all age group from LMICs. All stroke types [ischemic vs haemorrhagic stroke vs transient ischemic stroke] will be included. In-patient stroke participants in all care structure concern such as hospital, and health centers in LMICs will be included. General medical admission and studies from HIC will be excluded. LMICs are countries defined by World Bank Group [16]

Intervention/exposure

Exposure is the COVID-19 pandemic

For peer review only - http://bmjopen.bmj.com/site/about/guidelines.xhtml

Comparators

The study will compare rates of stroke admissions and case fatality between pre-COVID-19 pandemic era and peri/post-COVD-19 pandemic era.

Outcome of interest

The outcome of interest will be; (1) the impact of COVID-19 pandemic on ASA in LMICs. (2) the impact of COVID-19 pandemic on stroke CFR in LMICs.

Data source and search strategies

Four international databases [i.e., PubMed/Medline, Google Scholar, African Journals Online (AJOL), Latin American and Caribbean Health Sciences Literature (LILACS)] and one preprint database [medRxiv] will be searched for relevant studies on the impact of COVID-19 pandemic on stroke admissions and/or stroke case fatality in LMICs from December, 2019 to December,2021 without language restriction.

Medical Sub-Heading (MeSH) terms and free text will be used in the search approach. These terms will be combined with the Boolean operators 'OR' and 'AND'. The key term includes; Covid-19, Stroke, cerebrovascular accident, admissions, case fatality, Low- and middle-income countries, Africa, Sub-Saharan Africa, Asia, Latin America, Caribbeans. The full search string is shown in **supplementary file 1**

Other sources

The authors will evaluate the reference lists of papers that meet the inclusion and exclusion criteria to see whether there will be any additional studies that are luckily to be missed by our electronic search.

For peer review only - http://bmjopen.bmj.com/site/about/guidelines.xhtml

Data screening and selection

MA and LA will independently review the titles and abstracts against the pre-specified eligibility criteria for inclusion in the review, and then repeat the process after retrieving the full-text. A disagreement will be resolved by discussion or reference to a third reviewer

Data extraction and management

The data extraction will be done by two researchers independently using a pretested and standardized excel spreadsheet[17]. Discrepancies arising during abstract and full text review will be discussed and agreement reached by adjudication of a third author. Data such as the author's name, year of publication, country, study design, patients' characteristics {i.e., sex, age], pre-pandemic stroke admission and case fatality, peri-pandemic stroke admission and case fatality, total sample size will be extracted. Mendeley will be used to managed and remove duplicated articles.

Addressing missing data

If any of the selected articles contain inadequate information, we will contact the appropriate author through email to request missing data. If that is not possible, the data will be deleted. Furthermore, the authors will obtain raw data from the primary study authors wherever possible so that we can extract the missing data

Quality assessment and risk of bias

Joanna Briggs critical appraisal checklist for analytical cross-sectional studies will be used for the quality assessment and risk of bias[18]. The checklist's purpose is to assess the methodological quality of each study that will be included in the review. In systematic reviews, it is one of the most extensively utilized appraisal tools. In addition, the instrument was chosen because of its

For peer review only - http://bmjopen.bmj.com/site/about/guidelines.xhtml

objectivity and ease of use. The tools consist of eight questions with the following answers; yes, no, unclear, and not applicable with correct and rigorous methodology assigned yes responses. The quality assessments and bias risk assessments will be carried out by two independent peer reviewers. Discrepancies discovered during the abstract and full text reviews will be explored and resolved through discussion. To reduce selection bias, reviewers will be blinded to the article's author, journal, and year of publication during the title and abstract review.

Data synthesis

Extracted data will be exported into Stata (version 16; Stata Cooperation, TX, USA) from Microsoft excel 2013 for all analyses. The study selection procedure will be summarized using the 2020 PRISMA flowchart[19] (Figure 1). The characteristics of the studies will be presented and summarized in a Table. We aim to perform meta-analyses on pooled proportional change in ASA and CFR with 95%CI using a random-effects model. With regards to case fatality, inpatient, 7days, 14 days, ad 30 days CFR will be pooled where possible. The heterogeneity of effect size estimates across these studies will be quantified using the I^2 statistic and Q statistic's P-value[20]. Publication bias will be assessed using funnel plot and Egger's regression test if ≥10 studies are involved.

Where significant heterogeneity exists in the included studies, a sub group analysis will be performed to determine the sources of heterogeneity based on the following, Economic Status (Low-income country and Middle-income country), Continent (Africa, South America, Asia], Stroke type [ischemic vs haemorrhagic], study type [prospective vs retrospective, national/regional vs single hospital], and sample size [≤400 vs>400]. Leave one out sensitivity analysis will be performed to examine the impact of a single study on the overall pool estimat

For peer review only - http://bmjopen.bmj.com/site/about/guidelines.xhtml

Acknowledgement

None

Contributors

MA conceived the study, drafted the manuscript, critically revised the manuscript for methodological, and intellectual content. MGS drafted the manuscript, critically revised the manuscript for methodological and intellectual content. LA critically revised the manuscript for methodological and intellectual content. HB drafted the manuscript, critically revised the manuscript for methodological and intellectual content. COY drafted the manuscript, critically revised the manuscript for methodological and intellectual content. All authors approved the final manuscript. MA is the guarantor of the review

Funding

The authors have not declared a specific grant for this research from any funding agency in the public, commercial or not-for-profit sectors

Competing interest

The authors declare that there are no conflicts of interest

Patient consent for publication: Not required

Provenance and peer review

Not commissioned; externally peer-reviewed.

Data availability statement

For peer review only

All relevant data are within the manuscript

Reference

1. Zhao J, Li H, Kung D, Fisher M, Shen Y, Liu R. Impact of the COVID-19 Epidemic on Stroke Care and Potential Solutions. *Stroke*. 2020;(July):1996-2001. doi:10.1161/STROKEAHA.120.030225

2. WHO. *WHO. WHO African Region Numbers at a Glance. 2020; Available from: Https://Www.Afro.Who.Int/Health-Topics/Coronavirus-Covid-19.*; 2021.

3. Markus HS, Brainin M. COVID-19 and stroke—A global World Stroke Organization perspective. *Int J Stroke*. 2020;15(4):361-364. doi:10.1177/1747493020923472

4. Nannoni S, de Groot R, Bell S, Markus HS. Stroke in COVID-19: A systematic review and meta-analysis. *Int J Stroke*. 2021;16(2):137-149. doi:10.1177/1747493020972922

5. Siow I, Lee KS, Zhang JJY, Saffari SE, Ng A, Young B. Stroke as a Neurological Complication of COVID-19: A Systematic Review and Meta-Analysis of Incidence, Outcomes and Predictors: Stroke and COVID-19. *J Stroke Cerebrovasc Dis*. 2021;30(3):105549. doi:10.1016/j.jstrokecerebrovasdis.2020.105549

6. Alimohamadi Y, Tola HH, Abbasi-ghahramanloo A. Case fatality rate of COVID-19 : a systematic review and meta-analysis. *J PREV MED HYG*. 2021:311-320.

7. Dessie ZG, Zewotir T. Mortality‐related risk factors of COVID‐19 : a systematic review and meta‐analysis of 42 studies and 423 , 117 patients. *BMC Infect Dis*. 2021;21(855):1-28. doi:10.1186/s12879-021-06536-3

For peer review only - http://bmjopen.bmj.com/site/about/guidelines.xhtml

8. You Y, Niu Y, Sun F, et al. Impact of COVID- - 19 pandemic on haemorrhagic stroke admissions : a systematic review and meta- - analysis. *BMJ Open*. 2021;e050559:1-9. doi:10.1136/bmjopen-2021-050559

9. Reddy ST, Satani N, Beauchamp JES, et al. A meta-analysis of the global impact of the COVID-19 pandemic on stroke care & the Houston Experience. *Ann Clin Transl Neurol*. 2021;8(4):929–937. doi:10.1002/acn3.51322

10. Douiri A, Muruet W, Bhalla A, et al. Stroke Care in the United Kingdom During the. *Stroke*. 2021;(June):2125-2133. doi:10.1161/STROKEAHA.120.032253

11. Rudilosso S. Acute Stroke Care Is at Risk in the Era of. *Stroke*. 2020;(July):1-5. doi:10.1161/STROKEAHA.120.030329

12. Douiri A, Muruet W, Bhalla A, et al. Stroke Care in the United Kingdom during the COVID-19 Pandemic. *Stroke*. 2021;(June):2125-2133. doi:10.1161/STROKEAHA.120.032253

13. Gabet A, Grave C, Tuppin P, Chatignoux E, Béjot Y, Olié V. Impact of the COVID-19 pandemic and a national lockdown on hospitalizations for stroke and related 30-day mortality in France: A nationwide observational study. *Eur J Neurol*. 2021;28(10):3279-3288. doi:10.1111/ene.14831

14. Sarfo FS, Mensah NO, Opoku FA, Adusei-mensah N, Ampofo M, Ovbiagele B. COVID-19 and stroke: Experience in a Ghanaian healthcare system. *J Neurol Sci*. 2020;(January).

15. Moher D, Shamseer L, Clarke M, et al. Preferred reporting items for systematic review and meta-analysis protocols (PRISMA-P) 2015 statement. *Syst Rev*. 2015;(January):1-9.

For peer review only

16. Group WB. Data World Bank Country and Lending Groups. https://datahelpdesk.worldbank.org/knowledgebase/articles/906519-world-bank-country-and-lending-groups. Published 2019. Accessed September 29, 2021.

17. Ackah M, Yeboah CO, Ameyaw L. Risk factors for 30-day in-hospital mortality for in-patient with stroke in sub-Saharan Africa: Protocol for a systematic review and meta-analysis. *BMJ Open*. 2021;11(7):8-12. doi:10.1136/bmjopen-2021-049927

18. JBI. *Checklist for Analytical Cross Sectional Studies*.; 2017.

19. Page MJ, Mckenzie JE, Bossuyt PM, et al. The PRISMA 2020 statement : an updated guideline for reporting systematic reviews Systematic reviews and Meta-Analyses. *BMJ*. 2021. doi:10.1136/bmj.n71

20. Higgins JPT, Thompson SG. Quantifying heterogeneity in a meta-analysis. *Stat Med*. 2002;21(11):1539-1558. doi:10.1002/sim.1186

For peer review only - http://bmjopen.bmj.com/site/about/guidelines.xhtml

Figure legend

Figure 1: PRISMA 2020 flow diagram for new systematic reviews which included searches of databases, registers and other sources

For peer review only

For peer review only - http://bmjopen.bmj.com/site/about/guidelines.xhtml

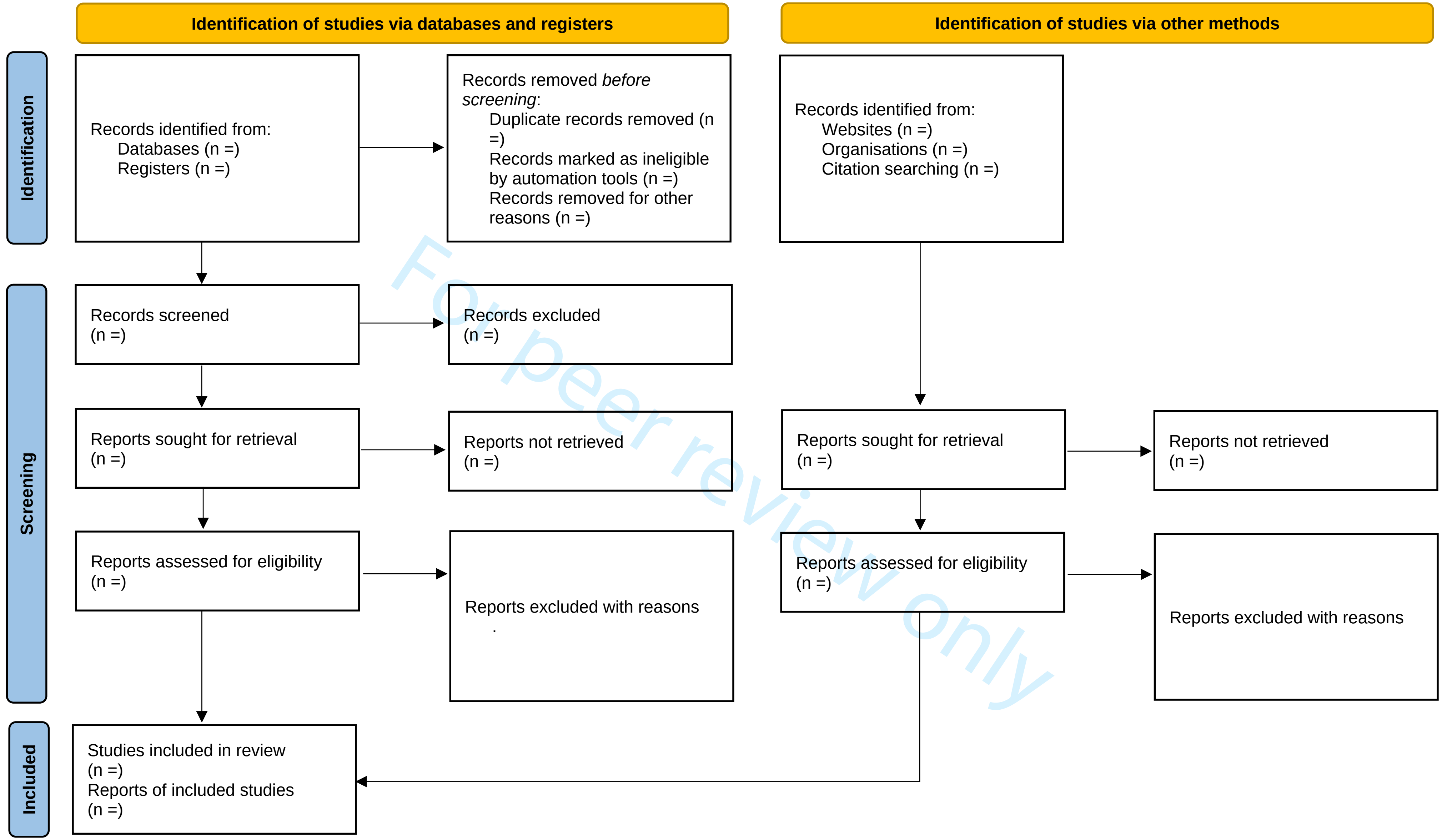

Figure 1: PRISMA 2020 flow diagram for new systematic reviews which included searches of databases, registers and other sources

For peer review only

Table 1: Search strings for electronic database

Search #	Searches
1.	Impact OR effect OR influence
2.	"COVID-19"[Mesh] OR "2019-nCoV"[Mesh] OR "severe acute respiratory syndrome coronavirus 2"[Mesh] OR "SARS-CoV-2"[Mesh] OR "Wuhan coronavirus" OR 2019 novel coronavirus* OR ‘’coronavirus disease 2019 virus’’* OR ‘’COVID19 virus’’* OR ‘’2019-nCoV infection’’ OR ‘’novel coronavirus pneumonia’’*
3.	Stroke OR cerebrovascular accident OR CVA OR cerebral infarction OR Ischemic stroke OR Lacuna stroke OR cerebral hemorrhage OR haemorrhagic stroke
4.	Mortality OR death OR case- fatality
5.	Asia OR Africa OR Caribbean OR ‘’Central America’’ OR ‘’South America’’
6.	low- and middle-income countr* OR low and middle income countr* OR low- and middle-income nation*OR low and middle income nation* OR low- and middle-income world OR low and middle income world OR low- and middle-income econom* OR low and middle income econom*OR low income countr* OR middle income countr* OR low-income countr* OR middle-income countr*OR low income nation* OR middle income nation* OR low-income nation* OR middle-income nation* OR low income world OR middle income world OR low-income world OR middle-income world OR low income econom*OR middle income econom* OR low-income econom* OR middle-income econom*
7.	((((((((Afghanistan* OR Benin* OR Burkina Faso* OR Burundi* OR Central African

For peer review only - http://bmjopen.bmj.com/site/about/guidelines.xhtml

	Republic* OR Chad* OR Comoros* OR Congo* OR Eritrea* OR Ethiopia* OR Gambia*OR Guinea-Bissau* OR Haiti* OR Korea Republic* OR Liberia* OR Madagascar* OR Malawi* OR Mali* OR Mozambique* OR Nepal* OR Niger* OR Rwanda* OR Sierra Leone* OR Somalia* OR South Sudan* OR Syrian Arab Republic* OR Tajikistan* OR Tanzania* OR Togo* OR Uganda* OR Yemen* OR Zimbabwe* OR Angola* OR Bangladesh* OR Bhutan* OR Bolivia* OR Cabo Verde* OR Cambodia* OR Cameroon*OR Congo* OR Ivory Coast* OR Djibouti* OR Egypt* OR El Salvador* OR Georgia* OR Ghana* OR Honduras* OR India* OR Indonesia* OR Kenya* OR Kiribati* OR Kosovo* OR Kyrgyz Republic* OR Lao PDP* OR Lesotho* OR Mauritania* OR Micronesia* OR Moldova* OR Mongolia* OR Morocco* OR Myanmar* OR Nicaragua* OR Nigeria* OR Pakistan* OR Papua New Guinea* OR Philippines* OR Sao Tome) and Principe*) OR Solomon Islands* OR Sri Lanka* OR Sudan* OR Swaziland* OR Timor-Leste* OR Tunisia* OR Ukraine* OR Uzbekistan* OR Vanuatu* OR Vietnam* OR West Bank) AND Gaza*) OR Zambia* OR Albania* OR Algeria* OR American Samoa* OR Armenia* OR Azerbaijan* OR Belarus* OR Belize* OR Bosnia) AND Herzegovina*) OR Botswana* OR Brazil* OR Bulgaria* OR China* OR Colombia* OR Costa Rica* OR Cuba* OR Dominica* OR Dominican Republic* OR Equatorial Guinea* OR Ecuador* OR Fiji* OR Gabon* OR Grenada* OR Guatemala* OR Guyana* OR Iran* OR Iraq* OR Jamaica* OR Jordan* OR Kazakhstan* OR Lebanon* OR Libya* OR Macedonia* OR Malaysia* OR Maldives* OR Marshall Islands* OR Mauritius* OR Mexico* OR Montenegro* OR Namibia* OR Nauru* OR Paraguay* OR Peru* OR Romania* OR Russian Federation* OR Samoa* OR Serbia* OR South Africa* OR Saint Lucia* OR Saint Vincent) AND the Grenadines*) OR Suriname* OR Thailand* OR Tonga* OR Turkey* OR Turkmenistan* OR Tuvalu* OR Venezuela*).mp.
8.	1 AND 2 AND 3 AND 4 AND 5 AND 7
9.	Limit 6 to humans

For peer review only - http://bmjopen.bmj.com/site/about/guidelines.xhtml

For peer review only

For peer review only - http://bmjopen.bmj.com/site/about/guidelines.xhtml

Checklist file 1: PRISMA-P (Preferred Reporting Items for Systematic review and Meta-Analysis Protocols) 2015 checklist: recommended items to address in a systematic review protocol

Section and topic	Item No	Checklist item	Page
**ADMINISTRATIVE INFORMATION**			
Title			
Identification	1a	Identify the report as a protocol of a systematic review	1
Update	1b	If the protocol is for an update of a previous systematic review, identify as such	NA
Registration	2	If registered, provide the name of the registry (such as PROSPERO) and registration number	2 & 6
Authors: contact	3a	Provide name, institutional affiliation, e-mail address of all protocol authors; provide physical mailing address of corresponding author	1
Authors' contribution	3b	Describe contributions of protocol authors and identify the guarantor of the review	11
Amendments	4	If the protocol represents an amendment of a previously completed or published protocol, identify as such and list changes; otherwise, state plan for documenting important protocol amendments	NA
Support			
Sources	5a	Indicate sources of financial or other support for the review	11
Sponsor	5b	Provide name for the review funder and/or sponsor	NA
Role of funder	5c	Describe roles of funder(s), sponsor(s), and/or institution(s), if any, in developing the protocol	NA
**INTRODUCTION**			
Rationale	6	Describe the rationale for the review in the context of what is already known	4-5
Objectives	7	Provide an explicit statement of the question(s) the review will address with reference to participants, interventions, comparators, and outcomes (PICO)	5
**METHODS**			
Eligibility criteria	8	Specify the study characteristics (such as PICO, study design, setting, time frame) and report characteristics (such as years considered, language, publication status) to be used as criteria for eligibility for the review	6-7
Information sources	9	Describe all intended information sources (such as electronic databases, contact with study authors, trial registers or other grey literature sources with planned dates of coverage	7

For peer review only

Search strategy	10	Present draft of search strategy to be used for at least one electronic database, including planned limits, such that it could be repeated	7
Study record			
Data management	11a	Describe the mechanism(s) that will be used to manage records and data throughout the review	8
Selection process	11b	State the process that will be used for selecting studies (such as two independent reviewers) through each phase of the review (that is, screening, eligibility and inclusion in meta-analysis)	8
Data collection process	11c	Describe planned method of extracting data from reports (such as piloting forms, done independently, in duplicate), any processes for obtaining and confirming data from investigators	9
Data items	12	List and define all variables for which data will be sought (such as PICO items, funding sources), any preplanned data assumptions and simplifications	-
Outcome and prioritization	13	List and define all outcomes for which data will be sought, including prioritization of main and additional outcomes, with rationale	7
Risk of bias in the individual studies	14	Describe anticipated methods for assessing risk of bias of individual studies, including whether this will be done at the outcome or study level, or both; state how this information will be used in data synthesis	9
Data synthesis	15a	Describe criteria under which study data will be quantitatively synthesized	9
	15b	If data are appropriate for quantitative synthesis, describe planned summary measures, methods of handling data and methods of combining data from studies, including any planned exploration of consistency (such as I^2, Kendall's τ)	
	15c	Describe any proposed additional analyses (such as sensitivity or subgroup analyses, meta-regression)	9
	15d	If quantitative synthesis is not appropriate, describe the type of summary planned	9
Meta biases	16	Specify any planned assessment of meta-bias(es) (such as publication bias across studies, selective reporting within studies)	9
Confidence in cumulative evidence	17	Describe how the strength of the body of evidence will be assessed (such as GRADE)	-

From: Shamseer L, Moher D, Clarke M, Ghersi D, Liberati A, Petticrew M, et al. Preferred reporting items for systematic review and meta-analysis protocols (prisma-p) 2015: Elaboration and explanation. BMJ [Internet]. 2015;349(January):1–25. Available from: http://dx.doi.org/doi:10.1136/bmj.g7647

For peer review only

For peer review only

BMJ Open

Impact of COVID-19 pandemic on acute stroke admissions and case-fatality rate in Lower- and Middle-Income Countries: a protocol for systematic review and meta-analysis.

Journal:	*BMJ Open*
Manuscript ID	bmjopen-2021-057893.R2
Article Type:	Protocol
Date Submitted by the Author:	05-Apr-2022

Complete List of Authors:	Ackah, Martin; Korle Bu Teaching Hospital, Physiotherapy; University of Ghana, School of Public Health, Department of Epidemiology and Disease Control<br>Salifu, Mohammed Gazali; Ministry of Health, Monitoring and Evaluation Directorate; University of Ghana, School of Public Health, Department of Epidemiology and Disease Control<br>Ameyaw, Louise; University of Ghana, School of public Health, Department of Epidemiology and Disease Control<br>Boakye, Hosea; University of Ghana, School of Public Health, Department of Biological, Environmental, and Occupational Health<br>Yeboah, Cynthia Osei; Korle Bu Teaching Hospital, Department of Physiotherapy

**Primary Subject Heading**:	Global health
Secondary Subject Heading:	Cardiovascular medicine, Epidemiology, Infectious diseases, Neurology, Public health
Keywords:	COVID-19, NEUROLOGY, Stroke < NEUROLOGY, EPIDEMIOLOGY

SCHOLARONE™ Manuscripts

Impact of COVID-19 pandemic on acute stroke admissions and case-fatality rate in Lower- and Middle-Income Countries: a protocol for systematic review and meta-analysis.

Martin Ackah[1, 3], Mohammed Gazali Salifu[2,3], Louise Ameyaw[3], Hosea Boakye[4], Cynthia Osei Yeboah[1]

1. Department of Physiotherapy, Korle Bu Teaching Hospital, Accra, Ghana
2. Monitoring and Evaluation Directorate, Ministry of Health, Accra, Ghana.
3. Department of Epidemiology and Disease Control, School of Public Health, University of Ghana, Accra, Ghana
4. Department of Biological, Environmental, and Occupational Health, School of Public Health, University of Ghana Accra, Ghana

Correspondence to Martin Ackah; martinackah10@gmail.com

For peer review only

Abstract

Introduction. The current review primarily aims to ascertain the impact of Coronavirus Disease-19 (COVID-19) on stroke admission as well as stroke case-fatality in Low-Middle Income Countries (LMICs).

Methods and analysis: Four international databases [PubMed/Medline, Google Scholar, African Journals Online (AJOL), Latin American and Caribbean Health Sciences Literature (LILACS)] and one preprint database [medRxiv]. Studies will be included if they are conducted in LMICs, all stroke types without age and language restriction, from December, 2019 to 31 December,2021. Two authors will screen the titles and abstracts against the pre-specified eligibility criteria for inclusion in the review, and then repeat the process after retrieving the full-text. Joanna Briggs critical appraisal checklist for analytical cross-sectional studies will be used for the quality assessment and risk of bias by two co-authors. The characteristics of the studies will be presented and summarized in a Table. We aim to perform meta-analyses on a pooled proportional change in acute stroke admissions and case-fatality with 95%CI using a random-effects meta-analysis. Publication bias will be assessed using funnel plot and Egger's regression test if ≥10 studies are involved. A sub group analysis will be performed to determine the sources of heterogeneity. Leave one out sensitivity analysis will be performed to examine the impact of a single study on the overall pool estimate.

Ethics and dissemination: Ethical approval is not required as this is secondary research and will use reported data in scientific literature. A full manuscript will be submitted to a reputable peer-review journal for publication and disseminated electronically and in print.

PROSPERO registration number: CRD42021281580

For peer review only - http://bmjopen.bmj.com/site/about/guidelines.xhtml

Strength ad limitation of this study

- This is the first systematic review and meta-analysis on the impact of Covid-19 pandemic on Acute Stroke Admission (ASA) and Case-fatality Rate (CFR) in LMICs
- A well-validated systematic review and meta-analysis methods that are fully compliant with established international norms and guidelines would be followed.
- To examine the robustness of the derived estimates, a sensitivity and sub-group analysis would be done.
- There would be no language restriction
- As a result of the regional and cultural variances, there would be significant heterogeneity across the studies.

Keywords: Impact; Stroke admission; Case fatality; COVID-19; LMICs

For peer review only - http://bmjopen.bmj.com/site/about/guidelines.xhtml

Introduction

The World Health Organization (WHO) announced a Severe Acute Respiratory Syndrome Coronavirus-2 (SARS Cov-2) pandemic in March 2020, posing a threat to healthcare systems and cultures around the world [1]. Since the first recorded case in December of 2019, more than 240 million Coronavirus Disease-19 cases and 4.7 million deaths have been confirmed worldwide as of September 25, 2021, with Africa accounting for approximately 3% of global incidence[2]

COVID-19 is largely a respiratory infection, but research in patients with severe infections has showed that it is a multi-system inflammatory condition with neurologic effects such as stroke [3]. For example, stroke rates among confirmed and hospitalized COVID-19 patients have been found to range from 1.4% to 1.74% according to recent global systematic review and meta-analyses [4,5].In 2021, the global predicted Case Fatality Rate (CFR) of COVID-19 ranged from 10%[6] to nearly 18%[7].

There have been conflicting accounts on the impact of Acute Stroke Admission (ASA) appearing in emergency rooms. For example, a systematic review and meta-analysis showed that the proportion of haemorrhagic stroke admissions increased in the pandemic period compared to the proportion of haemorrhagic stroke admission in the pre-pandemic era by 10%[8]. The study was limited by several factors; first, the total number of admissions during the pandemic was not reported, rendering the incidence of stroke inconclusive; second, the lack of data about stroke severity, ischemic stroke sub types, and prognoses, might have underestimated/overestimated the pandemic's impact on stroke to some extent[8]. Conversely, a global systematic review and meta-analysis on the impact of COVID-19 pandemic on ASA found a significant reduction of 29% compared to pre-pandemic period[9]. The authors did not conduct a sub-group analysis to determine the influence of the pandemic on ASA in LMICs. Furthermore, just a few papers from LMICs

For peer review only - http://bmjopen.bmj.com/site/about/guidelines.xhtml

were included in the prior review. Obviously, this cannot be generalized to represent the panoramic view of COVID-19 impact of ASA in LMICs.

Despite documented high-quality services with respect to workflow metrics, angiographic results, complications, outcomes at discharge, maintenance of reperfusion therapies as well as infection control measures[10,11], the pandemic has had a negative impact on stroke CFR in both high income countries and LMICs based on observational studies[12–14]. For instance, stroke CFR increased by 2.4 percentage points in the United Kingdom [12], and by a significant 60 percent in France [13]. A cross sectional study in SSA found an estimated peri-pandemic high stroke case fatality rates of 29.3% [14].

Several primary investigations have been carried out to determine the peri-pandemic stroke CFR in LMICs with widely varying reported magnitudes. The authors are unaware of any previous systematic reviews and/or meta-analyses on the subject in LMICs. With this in mind, the authors sort to conduct a systematic review and meta-analysis on the impact of COVID-19 pandemic on acute stroke hospitalization and death in limited-resource countries in the world.

Research questions

1. What is the impact of COVID-19 pandemic on ASA in LMICs?
2. What is the impact of the COVID-19 pandemic on post-stroke CFR in LMICs?

For peer review only

Method and analysis

Best practices and registration

The current protocol was registered in the PROSPERO database under the identifier CRD42021281580, and it was reported in compliance with Preferred Reporting Items for Systematic Review and Meta-analyses Protocol (PRISMA-P)[15] [checklist file]. The study will start on 30th April, 2022 and end on 30th August, 2022. A final paper will be published in a reputable peer review journal.

Eligibility criteria

Studies

Observational studies such as longitudinal, cohort, case control and cross-sectional studies reporting the impact of COVID-19 pandemic on stroke admission and case fatality in LMICs will be considered in the current review. Case series, case reports, narrative and systematic reviews, editorials, comments, letters, opinion pieces, abstracts, conference proceedings, and animal studies will all be excluded.

Participants

We aim to include impact of COVID-19 pandemic on ASA and CFR studies involving all age group from LMICs. All stroke types [ischemic vs haemorrhagic stroke vs transient ischemic stroke] will be included. In-patient stroke participants in all care structure concern such as hospital, and health centers in LMICs will be included. General medical admission and studies from HIC will be excluded. LMICs are countries defined by World Bank Group [16]

Intervention/exposure

Exposure is the COVID-19 pandemic

For peer review only - http://bmjopen.bmj.com/site/about/guidelines.xhtml

Comparators

The study will compare rates of stroke admissions and case fatality between pre-COVID-19 pandemic era and peri/post-COVD-19 pandemic era.

Outcome of interest

The outcome of interest will be; (1) the impact of COVID-19 pandemic on ASA in LMICs. (2) the impact of COVID-19 pandemic on stroke CFR in LMICs.

Data source and search strategies

Four international databases [i.e., PubMed/Medline, Google Scholar, African Journals Online (AJOL), Latin American and Caribbean Health Sciences Literature (LILACS)] and one preprint database [medRxiv] will be searched for relevant studies on the impact of COVID-19 pandemic on stroke admissions and/or stroke case fatality in LMICs from December, 2019 to December,2021 without language restriction.

Medical Sub-Heading (MeSH) terms and free text will be used in the search approach. These terms will be combined with the Boolean operators 'OR' and 'AND'. The key term includes; COVID-19, Stroke, cerebrovascular accident, admissions, case fatality, Low- and middle-income countries, Africa, Sub-Saharan Africa, Asia, Latin America, Caribbeans. The full search string is shown in **supplementary file 1**

Other sources

The authors will evaluate the reference lists of papers that meet the inclusion and exclusion criteria to see whether there will be any additional studies that are luckily to be missed by our electronic search.

For peer review only - http://bmjopen.bmj.com/site/about/guidelines.xhtml

Data screening and selection

Two independent authors will independently review the titles and abstracts against the pre-specified eligibility criteria for inclusion in the review, and then repeat the process after retrieving the full-text. A disagreement will be resolved by discussion or reference to a third reviewer

Data extraction and management

The data extraction will be done by two researchers independently using a pretested and standardized excel spreadsheet[17]. Discrepancies arising during abstract and full text review will be discussed and agreement reached by adjudication of a third author. Data such as the author's name, year of publication, country, study design, patients' characteristics [i.e., sex, age], pre-pandemic stroke admission and case fatality, peri-pandemic stroke admission and case fatality, total sample size will be extracted. Mendeley will be used to managed and remove duplicated articles.

Addressing missing data

If any of the selected articles contain inadequate information, we will contact the appropriate author through email to request missing data. If that is not possible, the data will be deleted. Furthermore, the authors will obtain raw data from the primary study authors wherever possible so that we can extract the missing data

Quality assessment and risk of bias

Joanna Briggs critical appraisal checklist for analytical cross-sectional studies will be used for the quality assessment and risk of bias[18]. The checklist's purpose is to assess the methodological quality of each study that will be included in the review. In systematic reviews, it is one of the most extensively utilized appraisal tools. In addition, the instrument was chosen because of its

For peer review only - http://bmjopen.bmj.com/site/about/guidelines.xhtml

objectivity and ease of use. The tools consist of eight questions with the following answers; yes, no, unclear, and not applicable with correct and rigorous methodology assigned yes responses. The quality assessments and bias risk assessments will be carried out by two independent peer reviewers. Discrepancies discovered during the abstract and full text reviews will be explored and resolved through discussion. To reduce selection bias, reviewers will be blinded to the article's author, journal, and year of publication during the title and abstract review.

Data synthesis

Extracted data will be exported into Stata (version 16; Stata Cooperation, TX, USA) from Microsoft excel 2013 for all analyses. The study selection procedure will be summarized using the 2020 PRISMA flowchart[19] (Figure 1). The characteristics of the studies will be presented and summarized in a Table. We aim to perform meta-analyses on pooled proportional change in ASA and CFR with 95%CI using a random-effects model. With regards to case fatality, inpatient, 7days, 14 days, ad 30 days CFR will be pooled where possible. The heterogeneity of effect size estimates across these studies will be quantified using the I^2 statistic and Q statistic's P-value[20]. Publication bias will be assessed using funnel plot and Egger's regression test if ≥10 studies are involved.

Where significant heterogeneity exists in the included studies, a sub group analysis will be performed to determine the sources of heterogeneity based on the following, Economic Status (Low-income country and Middle-income country), Continent (Africa, South America, Asia], Stroke type [ischemic vs haemorrhagic], study type [prospective vs retrospective, national/regional vs single hospital], and sample size [≤400 vs>400]. Leave one out sensitivity analysis will be performed to examine the impact of a single study on the overall pooled estimate.

Patients and public involvement

For peer review only - http://bmjopen.bmj.com/site/about/guidelines.xhtml

Patient or the public were not involved in the design, or conduct, or reporting, or dissemination plans of our research.

Ethics and dissemination

Ethical approval is not required as this is secondary research and will use reported data in scientific literature. A full manuscript will be submitted to a reputable peer-review journal for publication.

Acknowledgement

None

Reference

1. Zhao J, Li H, Kung D, Fisher M, Shen Y, Liu R. Impact of the COVID-19 Epidemic on Stroke Care and Potential Solutions. *Stroke*. 2020;(July):1996-2001. doi:10.1161/STROKEAHA.120.030225

2. WHO. *WHO. WHO African Region Numbers at a Glance. 2020; Available from: Https://Www.Afro.Who.Int/Health-Topics/Coronavirus-Covid-19.*; 2021.

3. Markus HS, Brainin M. COVID-19 and stroke—A global World Stroke Organization perspective. *Int J Stroke*. 2020;15(4):361-364. doi:10.1177/1747493020923472

4. Nannoni S, de Groot R, Bell S, Markus HS. Stroke in COVID-19: A systematic review and meta-analysis. *Int J Stroke*. 2021;16(2):137-149. doi:10.1177/1747493020972922

5. Siow I, Lee KS, Zhang JJY, Saffari SE, Ng A, Young B. Stroke as a Neurological Complication of COVID-19: A Systematic Review and Meta-Analysis of Incidence, Outcomes and Predictors: Stroke and COVID-19. *J Stroke Cerebrovasc Dis*. 2021;30(3):105549. doi:10.1016/j.jstrokecerebrovasdis.2020.105549

For peer review only - http://bmjopen.bmj.com/site/about/guidelines.xhtml

6. Alimohamadi Y, Tola HH, Abbasi-ghahramanloo A. Case fatality rate of COVID-19 : a systematic review and meta-analysis. *J PREV MED HYG*. 2021:311-320.

7. Dessie ZG, Zewotir T. Mortality - related risk factors of COVID - 19 : a systematic review and meta - analysis of 42 studies and 423 , 117 patients. *BMC Infect Dis*. 2021;21(855):1-28. doi:10.1186/s12879-021-06536-3

8. You Y, Niu Y, Sun F, et al. Impact of COVID- - 19 pandemic on haemorrhagic stroke admissions : a systematic review and meta- - analysis. *BMJ Open*. 2021;e050559:1-9. doi:10.1136/bmjopen-2021-050559

9. Reddy ST, Satani N, Beauchamp JES, et al. A meta-analysis of the global impact of the COVID-19 pandemic on stroke care & the Houston Experience. *Ann Clin Transl Neurol*. 2021;8(4):929–937. doi:10.1002/acn3.51322

10. Douiri A, Muruet W, Bhalla A, et al. Stroke Care in the United Kingdom During the. *Stroke*. 2021;(June):2125-2133. doi:10.1161/STROKEAHA.120.032253

11. Rudilosso S. Acute Stroke Care Is at Risk in the Era of. *Stroke*. 2020;(July):1-5. doi:10.1161/STROKEAHA.120.030329

12. Douiri A, Muruet W, Bhalla A, et al. Stroke Care in the United Kingdom during the COVID-19 Pandemic. *Stroke*. 2021;(June):2125-2133. doi:10.1161/STROKEAHA.120.032253

13. Gabet A, Grave C, Tuppin P, Chatignoux E, Béjot Y, Olié V. Impact of the COVID-19 pandemic and a national lockdown on hospitalizations for stroke and related 30-day mortality in France: A nationwide observational study. *Eur J Neurol*. 2021;28(10):3279-

For peer review only - http://bmjopen.bmj.com/site/about/guidelines.xhtml

3288. doi:10.1111/ene.14831

14. Sarfo FS, Mensah NO, Opoku FA, Adusei-mensah N, Ampofo M, Ovbiagele B. COVID-19 and stroke: Experience in a Ghanaian healthcare system. *J Neurol Sci*. 2020;(January).

15. Moher D, Shamseer L, Clarke M, et al. Preferred reporting items for systematic review and meta-analysis protocols (PRISMA-P) 2015 statement. *Syst Rev*. 2015;(January):1-9.

16. Group WB. Data World Bank Country and Lending Groups. https://datahelpdesk.worldbank.org/knowledgebase/articles/906519-world-bank-country-and-lending-groups. Published 2019. Accessed September 29, 2021.

17. Ackah M, Yeboah CO, Ameyaw L. Risk factors for 30-day in-hospital mortality for in-patient with stroke in sub-Saharan Africa: Protocol for a systematic review and meta-analysis. *BMJ Open*. 2021;11(7):8-12. doi:10.1136/bmjopen-2021-049927

18. JBI. *Checklist for Analytical Cross Sectional Studies*.; 2017.

19. Page MJ, Mckenzie JE, Bossuyt PM, et al. The PRISMA 2020 statement : an updated guideline for reporting systematic reviews Systematic reviews and Meta-Analyses. *BMJ*. 2021. doi:10.1136/bmj.n71

20. Higgins JPT, Thompson SG. Quantifying heterogeneity in a meta-analysis. *Stat Med*. 2002;21(11):1539-1558. doi:10.1002/sim.1186

For peer review only

For peer review only - http://bmjopen.bmj.com/site/about/guidelines.xhtml

Contributors

MA conceived the study, drafted the manuscript, critically revised the manuscript for methodological, and intellectual content. MGS drafted the manuscript, critically revised the manuscript for methodological and intellectual content. LA critically revised the manuscript for methodological and intellectual content. HB drafted the manuscript, critically revised the manuscript for methodological and intellectual content. COY drafted the manuscript, critically revised the manuscript for methodological and intellectual content. All authors approved the final manuscript. MA is the guarantor of the review

Funding

The authors have not declared a specific grant for this research from any funding agency in the public, commercial or not-for-profit sectors

Competing interest

The authors declare that there are no conflicts of interest

Patient consent for publication: Not required

Provenance and peer review

Not commissioned; externally peer-reviewed.

Data availability statement

All relevant data are within the manuscript

For peer review only - http://bmjopen.bmj.com/site/about/guidelines.xhtml

Figure legend

Figure 1: PRISMA 2020 flow diagram for new systematic reviews which included searches of databases, registers and other sources

For peer review only

For peer review only - http://bmjopen.bmj.com/site/about/guidelines.xhtml

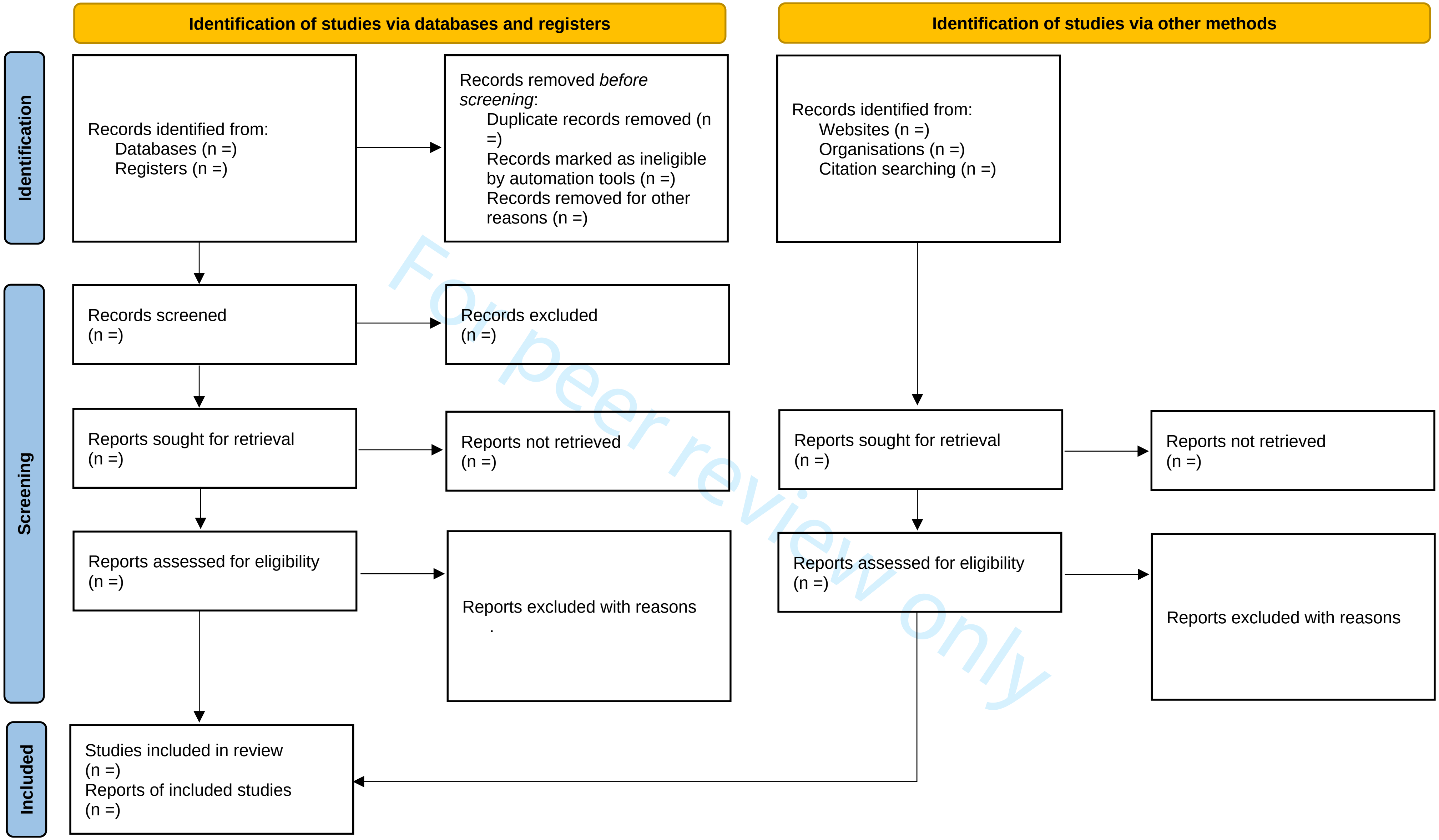

Figure 1: PRISMA 2020 flow diagram for new systematic reviews which included searches of databases, registers and other sources

Table 1: Search strings for electronic database

Search #	Searches
1.	Impact OR effect OR influence
2.	"COVID-19"[Mesh] OR "2019-nCoV"[Mesh] OR "severe acute respiratory syndrome coronavirus 2"[Mesh] OR "SARS-CoV-2"[Mesh] OR "Wuhan coronavirus" OR 2019 novel coronavirus* OR ‘’coronavirus disease 2019 virus’’* OR ‘’COVID19 virus’’* OR ‘’2019-nCoV infection’’ OR ‘’novel coronavirus pneumonia’’*
3.	Stroke OR cerebrovascular accident OR CVA OR cerebral infarction OR Ischemic stroke OR Lacuna stroke OR cerebral hemorrhage OR haemorrhagic stroke
4.	Mortality OR death OR case- fatality
5.	Asia OR Africa OR Caribbean OR ‘’Central America’’ OR ‘’South America’’
6.	low- and middle-income countr* OR low and middle income countr* OR low- and middle-income nation*OR low and middle income nation* OR low- and middle-income world OR low and middle income world OR low- and middle-income econom* OR low and middle income econom*OR low income countr* OR middle income countr* OR low-income countr* OR middle-income countr*OR low income nation* OR middle income nation* OR low-income nation* OR middle-income nation* OR low income world OR middle income world OR low-income world OR middle-income world OR low income econom*OR middle income econom* OR low-income econom* OR middle-income econom*
7.	((((((((Afghanistan* OR Benin* OR Burkina Faso* OR Burundi* OR Central African

For peer review only - http://bmjopen.bmj.com/site/about/guidelines.xhtml

	Republic* OR Chad* OR Comoros* OR Congo* OR Eritrea* OR Ethiopia* OR Gambia*OR Guinea-Bissau* OR Haiti* OR Korea Republic* OR Liberia* OR Madagascar* OR Malawi* OR Mali* OR Mozambique* OR Nepal* OR Niger* OR Rwanda* OR Sierra Leone* OR Somalia* OR South Sudan* OR Syrian Arab Republic* OR Tajikistan* OR Tanzania* OR Togo* OR Uganda* OR Yemen* OR Zimbabwe* OR Angola* OR Bangladesh* OR Bhutan* OR Bolivia* OR Cabo Verde* OR Cambodia* OR Cameroon*OR Congo* OR Ivory Coast* OR Djibouti* OR Egypt* OR El Salvador* OR Georgia* OR Ghana* OR Honduras* OR India* OR Indonesia* OR Kenya* OR Kiribati* OR Kosovo* OR Kyrgyz Republic* OR Lao PDP* OR Lesotho* OR Mauritania* OR Micronesia* OR Moldova* OR Mongolia* OR Morocco* OR Myanmar* OR Nicaragua* OR Nigeria* OR Pakistan* OR Papua New Guinea* OR Philippines* OR Sao Tome) and Principe*) OR Solomon Islands* OR Sri Lanka* OR Sudan* OR Swaziland* OR Timor-Leste* OR Tunisia* OR Ukraine* OR Uzbekistan* OR Vanuatu* OR Vietnam* OR West Bank) AND Gaza*) OR Zambia* OR Albania* OR Algeria* OR American Samoa* OR Armenia* OR Azerbaijan* OR Belarus* OR Belize* OR Bosnia) AND Herzegovina*) OR Botswana* OR Brazil* OR Bulgaria* OR China* OR Colombia* OR Costa Rica* OR Cuba* OR Dominica* OR Dominican Republic* OR Equatorial Guinea* OR Ecuador* OR Fiji* OR Gabon* OR Grenada* OR Guatemala* OR Guyana* OR Iran* OR Iraq* OR Jamaica* OR Jordan* OR Kazakhstan* OR Lebanon* OR Libya* OR Macedonia* OR Malaysia* OR Maldives* OR Marshall Islands* OR Mauritius* OR Mexico* OR Montenegro* OR Namibia* OR Nauru* OR Paraguay* OR Peru* OR Romania* OR Russian Federation* OR Samoa* OR Serbia* OR South Africa* OR Saint Lucia* OR Saint Vincent) AND the Grenadines*) OR Suriname* OR Thailand* OR Tonga* OR Turkey* OR Turkmenistan* OR Tuvalu* OR Venezuela*).mp.
8.	1 AND 2 AND 3 AND 4 AND 5 AND 7
9.	Limit 6 to humans

For peer review only

For peer review only

For peer review only - http://bmjopen.bmj.com/site/about/guidelines.xhtml

Checklist file 1: PRISMA-P (Preferred Reporting Items for Systematic review and Meta-Analysis Protocols) 2015 checklist: recommended items to address in a systematic review protocol

Section and topic	Item No	Checklist item	Page
**ADMINISTRATIVE INFORMATION**			
Title			
Identification	1a	Identify the report as a protocol of a systematic review	1
Update	1b	If the protocol is for an update of a previous systematic review, identify as such	NA
Registration	2	If registered, provide the name of the registry (such as PROSPERO) and registration number	2 & 6
Authors: contact	3a	Provide name, institutional affiliation, e-mail address of all protocol authors; provide physical mailing address of corresponding author	1
Authors' contribution	3b	Describe contributions of protocol authors and identify the guarantor of the review	11
Amendments	4	If the protocol represents an amendment of a previously completed or published protocol, identify as such and list changes; otherwise, state plan for documenting important protocol amendments	NA
Support			
Sources	5a	Indicate sources of financial or other support for the review	11
Sponsor	5b	Provide name for the review funder and/or sponsor	NA
Role of funder	5c	Describe roles of funder(s), sponsor(s), and/or institution(s), if any, in developing the protocol	NA
**INTRODUCTION**			
Rationale	6	Describe the rationale for the review in the context of what is already known	4-5
Objectives	7	Provide an explicit statement of the question(s) the review will address with reference to participants, interventions, comparators, and outcomes (PICO)	5
**METHODS**			
Eligibility criteria	8	Specify the study characteristics (such as PICO, study design, setting, time frame) and report characteristics (such as years considered, language, publication status) to be used as criteria for eligibility for the review	6-7
Information sources	9	Describe all intended information sources (such as electronic databases, contact with study authors, trial registers or other grey literature sources with planned dates of coverage	7

For peer review only

Search strategy	10	Present draft of search strategy to be used for at least one electronic database, including planned limits, such that it could be repeated	7
Study record			
Data management	11a	Describe the mechanism(s) that will be used to manage records and data throughout the review	8
Selection process	11b	State the process that will be used for selecting studies (such as two independent reviewers) through each phase of the review (that is, screening, eligibility and inclusion in meta-analysis)	8
Data collection process	11c	Describe planned method of extracting data from reports (such as piloting forms, done independently, in duplicate), any processes for obtaining and confirming data from investigators	9
Data items	12	List and define all variables for which data will be sought (such as PICO items, funding sources), any preplanned data assumptions and simplifications	-
Outcome and prioritization	13	List and define all outcomes for which data will be sought, including prioritization of main and additional outcomes, with rationale	7
Risk of bias in the individual studies	14	Describe anticipated methods for assessing risk of bias of individual studies, including whether this will be done at the outcome or study level, or both; state how this information will be used in data synthesis	9
Data synthesis	15a	Describe criteria under which study data will be quantitatively synthesized	9
	15b	If data are appropriate for quantitative synthesis, describe planned summary measures, methods of handling data and methods of combining data from studies, including any planned exploration of consistency (such as I^2, Kendall's τ)	
	15c	Describe any proposed additional analyses (such as sensitivity or subgroup analyses, meta-regression)	9
	15d	If quantitative synthesis is not appropriate, describe the type of summary planned	9
Meta biases	16	Specify any planned assessment of meta-bias(es) (such as publication bias across studies, selective reporting within studies)	9
Confidence in cumulative evidence	17	Describe how the strength of the body of evidence will be assessed (such as GRADE)	-

From: Shamseer L, Moher D, Clarke M, Ghersi D, Liberati A, Petticrew M, et al. Preferred reporting items for systematic review and meta-analysis protocols (prisma-p) 2015: Elaboration and explanation. BMJ [Internet]. 2015;349(January):1–25. Available from: http://dx.doi.org/doi:10.1136/bmj.g7647

For peer review only

For peer review only
